# Legal Notes for Institutional Workers

**Published:** 1916-02-12

**Authors:** 


### LEGAL NOTES FOR INSTITUTIONAL WORKERS.

### A Hardship of Scottish Law.

A bench of seven judges have just issued judgment on a point of law of general interest, and of particular importance to public health institutions. The action was one against the city of Glasgow for damages sustained by the plaintiff through being knocked down by a motor ambulance belonging to defendants, which at the time of the accident was conveying a patient to one of the city fever hospitals. The action was raised more than two months after the date of the accident, and the defendants pleaded that by Section 166 of the Public Health (Scotland) Act, 1897, the plaintiff's case could not now be heard, as that section provided that every action or prosecution against any person acting under that Act should be commenced within two months after the cause of action had arisen. In the Outer House Lord Ormidale had repelled that plea, and on the case coming on appeal to the Inner House it was sent for debate before a full bench for the purpose of uniformly settling so important a question of procedure. The result now is that the judgment of the Court below has been unanimously recalled, on the ground that the wrong alleged to have been done to plaintiff was done in execution of the defendants' duties under the Public Health Act, and, therefore, the section applied so as to exclude the action. The decision is one of much value to public health authorities; but we confess to sympathy with the plaintiff. In our law at present there is, we submit, sufficient protection to public bodies from vexatious litigation in the Public Authorities Protection Act, which compels a claimant to take steps to enforce his rights in Court within six months from their emergence. The framers of the Public Health Act might, in our opinion, have been well content to accept that limitation also. A two-months' limit was, however, inserted, and the statute was passed in that form. It is too short a period to allow an intending plaintiff, who might, for example, have been severely injured in an accident, to recover and get his case prepared so as to go into court. Doubtless his advisers ought to know that a short limit of time is the law; but it is only by chance that even a solicitor with the most varied practice could know that such an excluding provision was hidden in a statute specially applicable to public health. The provision and the judicial interpretation now put upon it are valuable to defendants under the Public Health Act; but they are scarcely just to the general public. Steps should be taken to repeal this Section.

### Tuberculosis as a Bar to Marriage.

A point decided in Jefferson *v.* Paskell, 140 L.T.J., page 7, should give satisfaction to eugenists. The action was for breach of promise of marriage, and the main defence was that the plaintiff was suffering from tuberculosis—an allegation which the jury, in fact, negatived. It was argued for the plaintiff that ill-health is not a justification for refusal to perform the promise to marry; and, dealing with that contention, Lord Justice Phillimore said: "On principle it would seem that there must be some cases of mental or physical infirmity, as it has been decided that there are cases of moral infirmity, which, supervening after the promise or first coming to the notice of the party after the promise, will justify him or her in refusing to marry." And Lord Justice Pickford said: "I think there may be tuberculosis existing to such an extent and of such a nature as to make a party unfit for marriage, and therefore afford a defence." Commenting on these *dicta* the journal referred to says: "It is surely abhorrent to common sense that a man who discovers after an engagement to marry that his *fiancée* is suffering from incurable illness should be compelled either to marry her or to pay damages, and *vice versa* in the case of a woman."

